# Neuromyelitis optica spectrum disorder: Exploring the diverse clinical manifestations and the need for further exploration

**DOI:** 10.1002/brb3.3644

**Published:** 2024-08-12

**Authors:** Hamid Noori, Mohammed Dheyaa Marsool Marsool, Krutika Mahendra Gohil, Muhammad Idrees, Tushar Subash, Zainab Alazzeh, Priyadarshi Prajjwal, Hritvik Jain, Omniat Amir

**Affiliations:** ^1^ Nuffield Department of Clinical Neurosciences University of Oxford Oxford UK; ^2^ Al‐Kindy College of Medicine University of Baghdad Baghdad Iraq; ^3^ Hinduhridaysamrat Balasaheb Thackeray Medical College and Dr. Rustom Narsi Cooper Municipal General Hospital Mumbai India; ^4^ Lahore General Hospital Lahore Punjab Pakistan; ^5^ Medical College The Aga Khan University Karachi Pakistan; ^6^ College of Medicine Jordanian University of Science and Technology Irbid Jordan; ^7^ College of Medicine Bharati Vidyapeeth Deemed University Pune India; ^8^ All India Institute of Medical Sciences Jodhpur India; ^9^ Almanhal Academy for Science Manhal University Khartoum Sudan

**Keywords:** autoimmune, clinical features, neuromyelitis optica, neuromyelitis optica spectrum disorders, optic neuritis, transverse myelitis

## Abstract

**Background:**

Neuromyelitis optica spectrum disorder (NMOSD) is an autoimmune disorder characterized by inflammatory assaults on the central nervous system (CNS), particularly on the optic nerves and spinal cord. In recent years, a wider range of clinical manifestations of this complex disease have been observed, emphasizing the importance of gaining a more profound understanding beyond optic neuritis (ON) and transverse myelitis (TM).

**Current knowledge:**

This study explores the many clinical symptoms of NMOSD, including common and uncommon presentations. Distinctive aspects of ON, TM, and diencephalic/brainstem syndromes are examined, highlighting their unique characteristics in contrast to conditions such as multiple sclerosis. We also discuss extra‐CNS involvement, such as unusual signs, including muscle involvement, retinal injury, auditory impairment, and rhinological symptoms.

**Aims and objectives:**

Our study intends to highlight the wide range and complexity of NMOSD presentations, emphasizing the significance of identifying unusual symptoms for precise diagnosis and prompt management. The specific processes that contribute to the varied clinical presentation of NMOSD are not well understood despite existing information. This emphasizes the necessity for more study to clarify the mechanisms that cause different symptoms and discover new treatment targets for this complex autoimmune disorder.

**Conclusion:**

It is essential to acknowledge the complex and varied clinical manifestations of NMOSD to enhance diagnosis, treatment, and patient results. By enhancing our comprehension of the fundamental processes and investigating innovative therapeutic approaches, we may aim to enhance the quality of life for persons impacted by this illness.

## Graphical abstract

This study examines the diverse clinical presentations of NMOSD, contrasting them with multiple sclerosis, and includes extra‐CNS symptoms like muscle, retinal, auditory, and rhinological issues. Recognizing these varied symptoms is crucial for accurate diagnosis and timely treatment, highlighting the need for further research to understand the underlying mechanisms and discover new therapeutic targets.

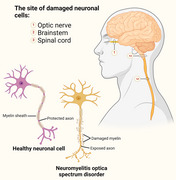

## 1 INTRODUCTION

Neuromyelitis optica spectrum disorder (NMOSD) is a severe autoimmune disease that involves inflammation and damage to the central nervous system (CNS), specifically impacting the optic nerves and spinal cord (Wingerchuk et al., 1999). Optic neuritis (ON) is a prominent characteristic of NMOSD, characterized by inflammation of the optic nerve, resulting in a decline in visual acuity and eye discomfort, eventually leading to blindness (Pula et al., 2014; Wingerchuk et al., 1999). This illness is more common among young patients and can lead to substantial vision loss or irreversible blindness. Research has identified specific features of ON in NMOSD that differentiate it from other illnesses like multiple sclerosis (MS) (Table 1) (Asgari et al., 2013). NMOSD‐associated ON often affects both eyes, is severe, and often leads to blindness (Kim et al., 2011). Profound visual impairment is a characteristic aspect of this condition. Furthermore, longitudinal investigations have demonstrated an increased susceptibility to blindness in people with NMOSD in comparison to individuals with MS‐related ON (Kim et al., 2011).

**TABLE 1:** Key distinguishing features of neuromyelitis optica spectrum disorder (NMOSD) and multiple sclerosis (MS).

Feature	NMOSD	MS
Autoantibody	Primarily AQP4‐positive	Target antigen unknown, presumably mediated driven by cell‐mediated response to myelin peptides
Demographics	More common in women (9:1), older age at onset	More frequent in females than males (female to male 3:1), with younger age at the onset
Clinical presentation	Primarily optic neuritis and longitudinally extensive transverse myelitis	Varied: relapsing‐remitting, primary‐progressive, etc.
Lesion location	Preferential involvement of optic nerves and spinal cord, longer spinal cord lesions	Widespread brain and spinal cord lesions, shorter spinal cord lesions
MRI findings	No Dawson's fingers, periventricular white matter lesions less common	Dawson's fingers, periventricular white matter lesions common
Prognosis	More severe relapses, absence of progressive phase between attacks, progression linked to accumulation of relapse‐associated disability	Variable course, relapses, and progression vary
Treatment	Primarily, immunosuppressive therapy with rituximab and azathioprine	Immunomodulatory and immunosuppressive therapy
	Novel biological agents like inebilizumab, ocrelizumab, tocilizumab, satralizumab, and eculizumab	Novel biological agents include ocrelizumab, masitinib, and ibudilast
Comorbidities	Increased risk of other autoimmune diseases	Less common

Abbreviations: AQP4, aquaporin‐4; MRI, magnetic resonance imaging.

In addition, NMOSD commonly presents with transverse myelitis (TM), which is defined by inflammation that spans three or more vertebral segments of the spinal cord. As a result, paralysis or sensory impairment occurs, which varies depending on the specific level of the spinal cord that is impacted (Huh et al., 2014). NMOSD‐related TM typically affects the central gray matter and has a tendency to reoccur, leading to a progressive accumulation of impairment (Huh et al., 2014). Aside from ON and TM, NMOSD can present with a range of uncommon neurological symptoms, such as diencephalic syndrome, brainstem syndrome, area postrema syndrome (APS), cerebral syndrome, hydrocephalus, lumbosacral myeloradiculitis, meningitis, and prenatal hydrocephalus (Chan et al., 2011).

Advanced imaging methods, such as magnetic resonance imaging (MRI), are essential for recognizing these uncommon manifestations, whereas aquaporin‐4 (AQP4) antibody testing assists in diagnosing NMOSD (Mealy et al., 2015). The treatment approaches for NMOSD generally consist of immunosuppressive medicines, such as corticosteroids, plasma exchange (PLEX), intravenous immune globulin (IVIG), and targeted monoclonal antibodies. Nevertheless, the effectiveness of these therapies fluctuates based on the particular clinical manifestation and the individual patient's reaction (Jacob et al., 2009). Current research endeavors persist in investigating the many expressions within the NMOSD spectrum and improving diagnostic criteria to enable prompt identification and focused therapy interventions.

In this study, we aim to comprehensively review the diverse clinical manifestations of NMOSD, including ON, TM, and rare neurological symptoms, to enhance understanding, diagnosis, and management strategies for this complex autoimmune condition.

## 2 TYPICAL FEATURES OF NMOSD

### 2.1 Optic neuritis

**FIGURE 1:**
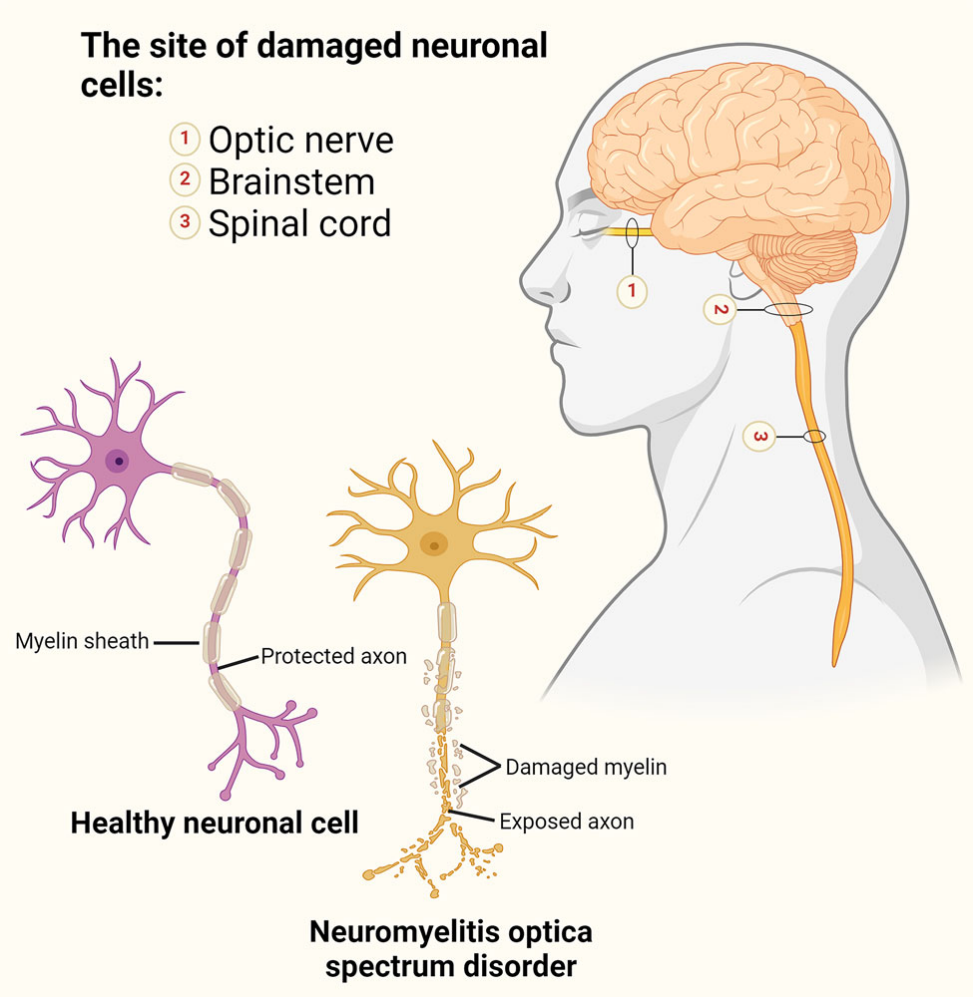
Most common sites affected by neuromyelitis optica spectrum disorder (NMOSD). NMOSD is primarily an astrocytopathic disease and can impact the optic nerve and spinal cord, which might result in symptoms such as visual impairments and discomfort or reduced strength in your extremities.

ON involves optic nerve inflammation progressing to the deterioration of visual acuity and ocular pain that can advance to blindness (Figure 1). ON occurs due to optic nerve damage among young individuals, which can induce debilitating vision impairment and/or permanent blindness. ON stands out as a distinctive highlight of NMOSD. Saini et al. (2010) deduced that optic nerve damage in NMOSD arises as a result of the greater AQP4 expression in the optic nerve compared to the brain, which heightens AQP4‐IgG binding. Following is the occurrence of complement‐mediated astrocyte damage, infiltration of granulocytes, oligodendrocyte death, and, ultimately, neuronal death, leading to optic nerve demyelination and damage.

Chen and Bhatti (2019) analyzed the features of ON in NMOSD, affirming that it tends to be bilateral, causes severe visual impairment, and frequently progresses to blindness with visual acuity <20/200. Roughly 50% of NMOSD patients exhibit isolated ON, with approximately 20% of these cases being bilateral. Wingerchuk et al. (1999) stated that sixty percent of NMOSD patients develop blindness around 7.7 years post‐onset, in contrast to only 4% of ON‐MS patients at 15‐year follow‐up. Beck et al. (1992) reported that profound visual loss is a hallmark of ON in NMOSD. Collongues et al. (2010) investigated the progression of visual acuity debilitation in pediatric patients as being more rapid than in adult patients. Optic nerve T2‐hyperintense lesion or T1‐weighted gadolinium‐enhancing lesion spreading over half of the optic nerve or involving the optic chiasma are the classic MRI findings seen. Fernandes et al. (2012) mentioned that ON‐NMOSD causes more severe visual field defects compared to ON‐MS. Anterior short‐segment inflammation is seen in ON‐MS compared to posterior long‐segment inflammation, which is seen in NMOSD. Optical coherence tomography showed increased loss of peripapillary retinal nerve fiber layer (RNFL) in superior and inferior quadrants in NMOSD, in contrast to the temporal RNFL loss that is typical of ON‐MS (Merle et al., 2008; Naismith et al., 2009). Pula et al. (2014) investigated that in acute ON, the enhancing anterior visual pathway lesion length >40 mm helps distinguish NMOSD from MS. This characteristic long involvement is called longitudinally extensive ON and is seen in NMOSD.

It is crucial to promptly and precisely distinguish between ON‐NMOSD and ON‐MS for proper treatment. MS misdiagnosis in patients with ON in keeping with NMOSD may lead to inappropriate use of MS‐modifying drugs and exacerbation of NMOSD (Kowarik et al., 2014). If an individual shows signs of bi‐temporal hemianopsia, longitudinal widespread optic nerve enhancement, or bilateral optic nerve involvement, it should prompt suspicion of ON‐NMOSD. Mealy et al. demonstrated that optic nerve enhancement of 17.6 mm or more had a sensitivity of 80.8% and specificity of 76.9%. The involvement of three or more optic nerve segments was 100% specific for ON‐NMOSD (Mealy et al., 2015). Several studies have shown evidence supporting the positive effects of PLEX in the acute treatment of ON‐NMOSD (Merle et al., 2012; Song et al., 2019).

### 2.2 Transverse myelitis

Central gray matter inflammation across three or more adjacent vertebral bodies often leads to paraplegia or quadriplegia, depending on the level of the spinal cord affected (Wingerchuk et al., 2006). Other clinical signs include severe itching caused by inflammation of itch fibers in the spinothalamic tract and repeated tonic spasms (Elsone et al., 2012; Usmani et al., 2012). Overall, 14% of patients with NMOSD may exhibit short spinal cord lesions that resemble those seen in MS (Flanagan et al., 2015). Intramedullary lesions that extend over three or more adjacent segments (longitudinally extensive transverse myelitis [LETM]) or three or more adjacent segments showing localized spinal cord atrophy in individuals with a history consistent with acute myelitis on MRI are indicative of LETM in NMOSD. LETM affecting the cervical part of the spinal cord may extend rostrally to the medulla.

Chee et al. (2018) found that in patients with LETM, the presence of cervico‐medullary junction involvement, a higher cord expansion ratio, bright spotty lesions, and being female significantly indicate a diagnosis of NMOSD. The cervical and upper thoracic spinal cord segments are more affected than the lower thoracic and lumbar regions due to the high concentration of AQP4 in the gray matter and glial cell processes near the ependymal cells of the central canal (Asgari et al., 2013; Kim et al., 2011). Asgari et al.’s MRI findings showed that LETM lesions transform into many shorter lesions after remission or treatment with high‐dose steroids (Kim et al., 2011). This process may lead to spinal cord atrophy due to recurrent myelitis, potentially causing neurological impairment. Cardiovascular autonomic dysfunction commonly occurs as a subsequent consequence of high‐thoracic spinal cord illness, leading to the development of autonomic dysreflexia characterized by fluctuating blood pressure and orthostatic hypotension in patients. Autonomic dysreflexia can develop in 7.5% of cases in the acute stage and up to 90% in the chronic stage after LETM (Jacob et al., 2009; Kulcu et al., 2009).

LETM can result in a spectrum of manifestations, including motor weakness, sensory impairment, and autonomic dysfunction. LETM episodes tend to recur, contributing to accumulating disability. In an acute attack, pulse steroid therapy is typically the primary mode of treatment. Alternative treatment choices like plasmapheresis, IVIG, rituximab, and eculizumab can be considered if the patient does not respond adequately to steroids (Huh et al., 2014; Matthews et al., 2013).

### 2.3 Diencephalic syndrome

NMOSD is primarily Astrocytopathic disease with secondary demyelination resulting in lesions not only in the optic nerves and spinal cord but also in the periventricular area where aquaporin four channels are prevalent. Diencephalic involvement in NMOSD represents a distinguishable aspect, extending beyond the more commonly diagnosed ON and TM. Lesions are seen around the third ventricles and cerebral aqueduct, involving the thalamus, hypothalamus, and front part of the midbrain. Possible clinical scenarios may involve syndrome of inappropriate antidiuretic hormone (SIADH) (O'Riordan et al., 1996), narcolepsy (Filippi et al., 1999), hypothermia, hypotension, excessive sleepiness, obesity (Kanbayashi et al., 2009), underactive thyroid, high levels of prolactin, absence of menstruation, milk secretion, and alterations in behavior (Whittam et al., 2017). AQP4 is highly expressed in the periventricular areas of the hypothalamus. Kanbayashi et al. (2009) documented narcolepsy with diencephalic lesions. In MS, diencephalic involvement is rare; however, in NMOSD, there is observed involvement in the hypothalamus, thalamic, and peri‐ependymal 3rd ventricle area (Jarius et al., 2008). Whittam et al. (2017) proposed that, in the presence of a diencephalic clinical syndrome, patients should be tested for AQP4 antibodies. Lesions involving the hypothalamic region can potentiate hyponatremia due to the SIADH (Lugaresi, 1992; Pittock et al., 2006) and, additionally, disrupt thermoregulation and hyperthermia (Nakashima et al., 2006). On MRI, a T2 hyperintense lesion in the thalamus and a Gd‐enhancing lesion of the hypothalamic parenchymal tissue are characteristic of the diencephalic syndrome of NMOSD. Hypothalamic lesions in NMOSD were documented in 5.0% of cases in the USA (Pittock et al., 2006), 5.3% in Japan (Nakashima et al., 2006), and 0% in Cuba (Cabrera‐Gómez et al., 2007). Hypothalamic lesions in MS are small and triangular or lobulated in shape, in contrast to the extensive lesions seen in NMOSD (Chan et al., 2011; Poppe et al., 2005; Qiu et al., 2011; Shinoda et al., 2011).

### 2.4 Brainstem syndrome

The dorsal part of the brainstem adjacent to the fourth ventricle is frequently affected in NMOSD. These lesions are abutting cervical lesions and occasionally occur independently without cervical involvement. The clinical vignette of brainstem syndrome includes nystagmus, dysarthria, dysphagia, ataxia, or ophthalmoplegia (Fung et al., 2012; Hage et al., 2011; Pittock & Lucchinetti, 2016). These lesions might either be the initial clinical sign of the illness or an acute worsening. Fung et al. (2012) have documented anorexia and weight loss in NMOSD patients with brainstem syndrome. In NMOSD, the most distinctive MRI anomaly is a lesion in the dorsal brainstem at the fourth ventricle, which involves the area postrema and the nucleus tractus solitarius (Popescu et al., 2011). Medullary lesions occur next to cervical cord lesions in a linear configuration. Acute brainstem syndrome overlaps with APS and includes individuals who exhibit oculomotor dysfunction or other cranial nerve palsies.

### 2.5 Area postrema syndrome

APS presents as intractable nausea, vomiting, and/or hiccoughs secondary to emetic reflex center inflammation in the rhomboid fossa of the 4th ventricle. APS is the first clinical manifestation of NMOSD in roughly 12% of cases (Apiwattanakul et al., 2010). Tissue rarefaction, blood vessel thickening, and preservation of myelin in the subependymal medullary tegmentum are found in area postrema lesions (Broadwell & Sofroniew, 1993). The medullary floor of the fourth ventricle and area postrema express AQP4 abundantly and, consequently, are targets for NMOSD lesions. In contrast to MS, APS is a characteristic of NMOSD and can occur as an isolated presentation of early disease (Popescu et al., 2011).

The area postrema features permeable endothelial cell connections, allowing brain cells in this region to be reached by circulating IgG. The area postrema is a highly vascularized brain region with a greater plasma flow and surface area/permeability ratio compared to nearby medullary areas (Kim et al., 2012). The delayed clearance of NMO IgG and endothelial permeation contribute to maintaining a preeminent concentration around astrocytic end feet, potentially heightening the susceptibility of the area postrema to AQP4 IgG‐induced pathophysiology. Both MRI and clinical evidence support the idea that the area postrema is a significant target in NMOSD patients, suggesting it serves as an entry portal for circulating IgG into the CNS (Asgari et al., 2013). Popescu et al. (2011) observed that area postrema astrocytes resist complement‐mediated lysis, which may progress to activate a cascade of inflammatory response that can be propagated throughout the CNS, thus enhancing BBB permeability and promoting AQP4 antibodies to exacerbate disease pathology.

APS is a definite entity in NMOSD. It can herald the typical ON or LETM from months to years (Apiwattanakul et al., 2010).

### 2.6 Cerebral syndrome

Cerebral involvement can be asymptomatic. However, it can also cause encephalopathy, seizures, and hemiparesis. AQP4 is expressed in the peri‐ependymal region. Recently, cortical oscillopsia without nystagmus‐related cortical involvement in a patient with AQP4‐Ab‐seropositive NMOSD was reported by Kim et al. (2012). Cerebral cortex involvement is rare. It has been reported previously in two previous studies (Kim et al., 2016; Song et al., 2019). The lesions influencing the cortex are located in the frontal, posterior parietal, and occipital lobes. Cortex‐involving lesions in fluid‐attenuated inversion recovery pictures showed diverse signal intensities and unclear boundaries, impacting both cortex and subcortex at the same time. Kim et al. (2012) found that aberrant brain MRI results in about 75% of patients, with cerebral cortex involvement present in just 3% of instances, emphasizing its rarity. Most patients with cortex involvement are young females who exhibit symptoms like headache, confusion, seizure, and focal deficits. Approximately half of these patients show cortical involvement at the initial presentation, as seen in the case reported by Ramineni et al. (2021).

## 3 RARE NEUROLOGICAL MANIFESTATIONS IN NMOSD

A preponderance of individuals with NMOSD exhibit MRI abnormalities in the brain, primarily localized to aquaporin 4‐rich circumventricular and periaqueductal regions (Pittock et al., 2006). However, the rare neurological manifestations of NMOSD present as a challenging subset of this complex spectrum, which requires vigilance and clinical acumen. Understanding the extensive distribution and purview of NMOSD, including its uncommon clinical presentations, is crucial to enabling an expeditious diagnosis when a patient exhibits such symptoms. Advanced imaging techniques like MRI are adopted for the identification of these rare presentations. Furthermore, testing for aquaporin 4 antibodies has become utilitarian in diagnosing individuals with these manifestations as patients of NMOSD.

### 3.1 Hydrocephalus

Ependymal cells lining the cerebral aqueduct express AQP4. Clardy et al. speculated that the binding of IgG antibodies to AQP4 initiates an inflammatory cascade of events. Consequently, scarring and stenosis of the cerebral aqueduct occur, thereby leading to obstruction of the channel (Clardy et al., 2014). Feng et al. reported a 9.6% incidence of severe spontaneous hydrocephalus post stenosis of the aqueductal channel in AQP4‐null mice. The authors also postulated that marked disorganization of the ependyma was displayed, and the rise in intracranial tension led to death within 6 weeks (Feng et al., 2009). AQP4 dysfunction occurring as a result of NMOSD can cause decreased cerebrospinal fluid (CSF) resorption, which can exacerbate hydrocephalus. Skjolding et al. (2013) performed a western blot study and deduced that AQP4 immunoreactivity was higher in the human hydrocephalic brain. Only three case reports of NMOSD presenting with hydrocephalus have been reported till now (Feng et al., 2009; Gratton & Mora, 2013; Rohatgi et al., 2023). The prolonged exposure of AQP4 to lymphocytes in encephalic regions could lead to the generation of autoantibodies, subsequently affecting the optic nerve, ciliary body, and aqueduct. This chain of events potentially instigates NMOSD and noncommunicating hydrocephalus. A comprehensive understanding and recognition of hydrocephalus in NMOSD is paramount for timely diagnosis and improvement of prognosis.

### 3.2 Lumbosacral myeloradiculitis

AQP4 is predominantly found in the gray matter and glial cell processes proximate to the ependymal cells of the central canal in the spinal cord (Kim et al., 2011). The inflammatory process of NMOSD often involves more than three vertebral segments, mainly involving central gray matter. These lesions appearing hyperintense on T2‐weighted images and hypointense on T1‐weighted images may correspond to a higher level of expression of AQP4 in medullary gray matter. Takai et al. reported two cases of lumbosacral myeloradiculitis in NMOSD. The authors described that the chronic demyelinating lesions seen at the right ventral portion of the lumbar cord exhibited loss of glial fibrillary acidic protein and AQP4, thereby confirming the diagnosis of NMOSD. The patient presented with gait disturbances, and the MRI scan demonstrated an enhancement in conus medullaris, spinal roots, and cauda equina. The authors speculated the CNS‐PNS transition zone is the target of radiculitis in NMOSD (Takai et al., 2012). The other case reported by Takai et al. revealed a lesion extending from Th11 to the conus medulla and involving the right side of the cord and cauda equina on spinal MRI. Post‐completing treatment with PLEXs, the patient did not show any additional exacerbations (Fadda et al., 2022). Fadda et al. (2022) concluded that conus medullaris involvement is seen in 10%–15% of patients with patchy or ring‐like gadolinium enhancement in more than 90% of cases.

### 3.3 Meningeal involvement

Wang et al. (2021) enumerated that a dysfunction in the meningeal lymphatics as a result of NMOSD progressed to meningeal inflammation. Meningitis is infrequently reported as the primary clinical manifestation in NMOSD (Shi et al., 2020). Bingxin et al. and Wang et al. reported that the AQP4‐antibody positive NMOSD patients developed signs of positive meningeal irritation as their initial presentation (Shi et al., 2020; Yaregal et al., 2021). Wang et al. described that headache with fever, meningeal irritation, and leukocytosis in the CSF with the cell count being more than 50 × 106/L in 13%–35% of patients and up to 1000 × 106/L in a few cases as the initial presentation broadens the NMOSD realm. Contrast‐enhanced MRI exhibited enhancement of bilateral periventricular parenchyma (cloud‐like enhancement) and bilateral ependyma (pencil‐thin enhancement), denoting typical NMOSD findings. Additionally, enhancement was seen in the meninges (Dutra et al., 2018; Wang et al., 2013). The absence of oligoclonal bands in the CSF contributes toward the diagnosis of NMOSD, with standard sensitivity and specificity. For the patient experiencing this subset of symptoms at first onset, the diagnosis poses a significant challenge. However, the alleviation of symptoms, based on the administration of glucocorticoids both clinically and on radiological assessment, is promising. Based on these features and the growing number of cases reported, meningeal involvement is considered an atypical phenotype within the NMOSD spectrum, and this requires careful differentiation from intracranial infection.

### 3.4 Fetal hydrocephalus

A case of severe neonatal hydrocephalus with permanent neurologic disability in a mother with NMOSD has been reported by Nour et al. The authors have speculated that this may be due to the AQP4 autoantibody crossing the placenta and inciting aqueductal inflammation (Nour et al., 2015).

## 4 OTHER ORGAN INVOLVEMENT NMOSD

AQP4 presence within multiple extracerebral organs has been verified with the possible hypothesis of its extra‐neural presentations in NMOSD. Briefly, the epithelium of salivary, olfactory, and lacrimal glands, retinal muller cells, organ of Corti in the inner ear, bronchiolar epithelium of lungs, basal cells of gastric mucosa, fast‐twitch cells of skeletal muscles, cells of renal collecting tubules, and placenta cells found consistent with AQP4 in variable molecular characteristics (Rosales & Kister, 2016). Lack of protective CD (complement decay) factors within CNS AQP4 as opposed to other organs like CD46 guards renal and skeletal AQP4 might be postulated as the theory of aggression in CNS than in other organ system. Saying other organ manifestations of NMOSD might be impossible without producing AQP4 as a pathological factor despite the aforementioned possibility (Asavapanumas et al., 2021).

### 4.1 Muscular manifestations

AQP4 loss from the sarcolemma with the presence of AQP4‐IgG, complement activation, and inflammatory cells may mark the muscular pathology of NMO. Both symptomatic and asymptomatic hyperCKemia could occur in NMO, as manifested in several clinical scenarios. A coinciding picture was reported in a seropositive AQP4‐IgG case of relapsing NMO complaining of myalgias with raised creatine kinase (CK) levels (Guo et al., 2014). We found high CK levels without muscular aches at both the initial and relapsing stages of NMO, targeting our hypothesis of AQP4 pathology in fast‐twitch muscular fibers (Deguchi et al., 2012). Significant hyperCKemia of 12520, 19415, and 59660 IU/L was assessed in patients of 7, 34, and 67‐years old females suffering from ON associated with generalized body aches, followed in a retrospective study by Suzuki et al. (2010). Furthermore, two diagnosed cases of NMO presented with reduced power and tender musculature of upper extremities found hyperCKemia, which revealed later low expression of AQP4 with complement saturation after biopsy (Malik et al., 2014). Four out of 43 patients with NMO elucidated hyperCKemia answering questionable association between raised CK and myopathy under the spectrum of NMOSD (Shouman et al., 2019).

### 4.2 Retinal injury

Water and electrolyte balance may be maintained by AQP4 on the muller cells of the retina. Activated complement module may disrupt AQP4 of muller cells, leading to macular edema as stated in a study (Netti et al., 2021). Various studies showed provident results of microcystic macular edema association with NMOSD, which is caused by ON damaging AQP4 on muller cells (Vujosevic et al., 2023).

IgG‐induced AQP4 demise, complement deposition, and resultant retinal injury might be the possible mechanism for this variant (Filippatou et al., 2021). A longitudinal study published congruent results of thinning of the ganglion and inner plexiform layer in patients without ON but positive AQP4‐IgG defeating the role of ON in retinal damage. Our saying of the direct role of AQP4‐IgG of NMOSD in the massacre of retinal layers might be supportive of such pathology (Oertel et al., 2018). A statistically significant decrease in the outer nuclear layer and average macular thickness without any change in the peripapillary RNFL in the population without ON further strengthened retinal damage in NMOSD by AQP4‐IgG directly (Akiba et al., 2016).

### 4.3 Auditory presentation

Knocking out AQP4 on the organ of Corti and cochlear nerve hampers its osmoregulatory function with resultant hearing loss might be the possible explanation for this rare presentation in NMOSD. Hearing loss could be due to brainstem lesions in NMOSD, but AQP4 instability was pointed secondary to antibody mediated inflammation rather than direct cellular damage. A case of cochlear‐type deafness showed significant improvement by managing with steroids and reducing AQP4‐IgG antibodies may foster inflammation‐mediated otologic damage as the basis of its background pathology in NMOSD. Delaying diagnosis and treatment in relapsing NMOSD may cause permanent loss and this must be noted with each follow‐up (Shaw et al., 2021). Another case of hearing loss was suspected secondary to a brainstem lesion but was later revealed as a member of NMOSD. In this case, maintenance steroids therapy with PLEXs reduced AQP4‐IgG with complete recovery of hearing loss. Such results may encourage diagnostic and prognostic perks, which must be valued while managing NMOSD (Onda et al., 2021).

Only 1.0% of 101 patients with NMOSD complained of sensorineural hearing loss in the Japanese study speaks about its peculiar proportion but it should not be neglected (Tanaka & Tanaka, 2016). Tugizova et al. (2020) presented two rare cases of deafness as a manifestation of relapsing NMOSD with seropositive AQP4‐IgG. Early recognition of such cases could prevent permanent hearing loss. Both types of deafness, conductive and sensorineural, could occur as auditory symptoms in NMOSD, as found in respective cases (Tugizova et al., 2020).

### 4.4 Rhinological manifestations

It is established that autoimmune mediated loss of AQP4 present on the glomeruli of the olfactory bulb leads to hyposmia (Jung et al., 2020). Immune‐mediated damage of the olfactory pathway in NMO may cause decreased olfaction found in previous literature. Overall, 51.4% of patients with NMO had dysregulated olfaction recently documented in a study emphasizing its significance in patients with NMOSD (Li et al., 2018). A statistical study highlighted that reduced olfactory bulb volume secondary to IgG‐mediated AQP4 loss was found in up to 53% of patients with NMOSD (Zhang et al., 2015). Hyposmia was the major complaint in 50% of patients with NMO relating to the significant impact of NMO on olfactory dysfunction. To repeat, disturbed olfaction might be included as a rare manifestation of NMOSD. It must be noticed in relapsing NMOSD as a peripheral entity for initiation of early treatment (Schmidt et al., 2013).

### 4.5 Placental presentation

The placenta being a vascular organ has abundant AQP4 on its syncytiotrophoblast and villi to regulate water and electrolytes between feto‐maternal tissue. Deletions of AQP4 in mice models increased the frequency of abortions due to necrosis may signify its role in human beings (Pérez‐Pérez et al., 2020). In pregnancy, AQP4 expression increases, which may enhance inflammatory response in NMOSD patients via AQP4‐IgG response, with a subsequent rise in the risk of miscarriages. Postpartum relapse of NMOSD has been experienced by such patients as well. Reactive auto‐antibodies may cross the placenta, exacerbating fetal preexisting autoimmune diseases (Mao‐Draayer et al., 2020). Preeclampsia risk may aggravate in a few NMOSD pregnant ladies but at the same time, a large percentage of abortions were highlighted in some studies. However, fetal death, stunted growth, or anomalies were not appreciated in such patients (Chang et al., 2018; D'Souza et al., 2020; Huda et al., 2019).

Contrasting results were also received from a study stating no significant under‐expression of AQP4 in patients with NMOSD and raised AQP4‐IgG antibody (Chandrasekar et al., 2022; Chang et al., 2018; Huda et al., 2019; Rosales & Kister, 2016).

## 5 THE BRIDGE BETWEEN MYASTHENIA GRAVIS AND NMOSD

Recently, a link between MG and NMOSD has been surfacing and has been reported in many cases. In most cases, however, thymectomy preceded the development of NMOSD, which likely predisposed the patients to autoimmune conditions. The thymus primarily serves an important function in building tolerance against self‐antigens and primarily by preventing the production of autoantibodies against self‐antigens, such as AQP4 protein, in the case of NMOSD. Thus, post‐thymectomy, due to the production of autoreactive T‐cells and loss of regulatory proteins, patients are likely to present with NMOSD. It is important to note that this may be true the other way as well, that is, patients with NMOSD may also present with MG (Kister et al., 2006; Vaknin‐Dembinsky et al., 2011).

## 6 ASSOCIATION OF NMOSD WITH OTHER AUTOIMMUNE DISORDERS

Apart from MG, NMOSD has been greatly associated with multiple systemic autoimmune disorders with a surge in non‐organ‐specific autoantibodies. For instance, a study by Chen et al. (2016) described how the probability of ANA and anti‐SSA antibodies was higher in the NMOSD group of patients. Thus, this shows that the trend of multiple autoantibodies and autoimmune disorders may be more common than was thought initially. Some other examples of such disorders include celiac disease, Sjogren's syndrome, systemic lupus erythematosus, and narcolepsy (Iyer et al., 2014).

## 7 NMOSD AS A PARANEOPLASTIC DISORDER

Paraneoplastic disorders may be defined as effects of the primary cancer and are often located in sites other than the location of the cancer. They are often the results of onconeural antigens that eventually trigger autoimmune reactions since they are considered foreign by the human body. As described earlier, AQP4 protein is found in a plethora of organs in our bodies and this makes it a potential onconeural antigen too, predisposing patients to develop NMOSD. The presence of a primary tumor in NMOSD, though a rare occurrence, has been reported to be higher than that in MS and may cause carcinomas of organs such as the breast, gut, thyroid, and bladder, and the list does not end here. Multiple such cases have been reported recently, supporting this hypothesis. For instance, Yang et al. (2014) presented a case of a patient with an invasive thymoma eventually developing ON and was positive for APQ4‐IgG and ANNA‐1 autoantibodies. In another study, Figuero et al. (2014) presented a case of a small‐bowel neuroendocrine tumor presenting with NMOSD as well and also positive for APQ4 antibodies. This shows that the extent of NMOSD with any tumor is not limited to any organ and may present with any neoplasm at any time.

## 8 CONCLUSION

NMOSD is characterized by various neurological symptoms and connections to organs outside the brain. The unique characteristics, such as severe ON and longitudinally widespread TM, highlight the significance of precise diagnosis and customized treatment. Identifying uncommon manifestations highlights the need for a thorough assessment. The connection between NMOSD and other autoimmune conditions, as well as its potential as a paraneoplastic illness, emphasizes its widespread effects. Ongoing research is essential for understanding the cause of NMOSD and creating specific treatments. Collaboration between doctors and researchers provides optimism for improving NMOSD care despite obstacles.

## AUTHOR CONTRIBUTIONS

**Hamid Noori**: Conceptualization; investigation; supervision; validation writing—original draft. **Mohammed Dheyaa Marsool Marsool**: Investigation; visualization; writing—original draft; writing—review and editing. **Krutika Mahendra Gohil**: Investigation; resources; validation; writing—original draft; writing—review and editing. **Muhammad Idrees**: Investigation; resources; validation; writing—original draft; writing—review and editing. **Tushar Subash**: Resources; validation; writing—original draft. **Zainab Alazzeh**: Conceptualization; investigation; validation; writing—original draft; writing—review and editing. **Priyadarshi Prajjwal and Hritvik Jain**: Validation; writing—original draft; writing ‐review and editing. **Omniat Amir**: Writing—review and editing.

## CONFLICT OF INTEREST STATEMENT

The authors declare no conflicts of interest, financial, or otherwise.

## FUNDING INFORMATION

None.

### PEER REVIEW

The peer review history for this article is available at https://publons.com/publon/10.1002/brb3.3644.

## GUARANTOR

Hamid Noori.

## Data Availability

Data sharing is not applicable to this article as no new data were created or analyzed in this study.
